# Sesamin Enhances Cholesterol Efflux in RAW264.7 Macrophages

**DOI:** 10.3390/molecules19067516

**Published:** 2014-06-06

**Authors:** Nan Liu, Chongming Wu, Lizhong Sun, Jun Zheng, Peng Guo

**Affiliations:** 1 Cardiac Surgical Intensive Care Unit, Beijing Anzhen Hospital, Capital Medical University, Beijing Institute of Heart Lung and Blood Vessel Diseases, Beijing 100029, China; E-Mail: ln9102@vip.sina.com; 2 Pharmacology and Toxicology Research Center, Institute of Medicinal Plant Development, Chinese Academy of Medical Sciences & Peking Union Medical College, Beijing 100094, China; E-Mail: wucm1979@gmail.com; 3 Department of Cardiovascular Surgery, Beijing Anzhen Hospital, Capital Medical University, Beijing Institute of Heart Lung and Blood Vessel Diseases, Beijing 100029, China; E-Mails: anzhen5@126.com (L.S.); dr.zhengjun@gmail.com (J.Z.)

**Keywords:** sesamin, atherosclerosis, foam cell, cholesterol efflux, macrophage

## Abstract

Foam cells formation as a result of the uncontrolled cytophagy of modified cholesterol by macrophages plays a key role in the occurrence and development of atherosclerosis. Sesamin is an active constituent of *Sesamum indicum* which has been shown to possess multiple pharmacological activities. In this work, we investigated the effects of sesamin on foam cell formation and cholesterol efflux in RAW264.7 macrophages. Sesamin dose-dependently inhibited the enhanced cholesterol accumulation elicited by oxidized low-density lipoprotein cholesterol (oxLDL) in RAW264.7 cells. Treatment with sesamin (10 μM) significantly enhanced cholesterol efflux mediated by high-density lipoprotein (HDL). Realtime quantitative PCR and luciferase assays showed that sesamin significantly increased the mRNA levels of PPARγ, LXRα, and ABCG1, and increased the transcriptional activity of PPARγ. The stimulating effect of sesamin on cholesterol efflux was substantially inhibited by the co-treatment with GW9662, a potent inhibitor of PPARγ. These results suggest that sesamin is a new inhibitor of foam cell formation that may stimulate cholesterol efflux through upregulation of the PPARγ-LXRα-ABCG1 pathway.

## 1. Introduction

The accumulation of cholesterol-laden macrophages (foam cells) in the arterial wall plays a key role in the occurrence and development of atherosclerosis [1,2]. The formation of foam cells is usually caused by either the uncontrolled cytophagy of modified low-density lipoprotein (LDL) or impaired cholesterol efflux [2,3], which is an indication of plaque-build up or atherosclerosis. Accumulating evidence demonstrates that reverse cholesterol transport (RCT) is a good antiatherogenic strategy by which excess cholesterol is transported from the peripheral cells back to the liver for clearance into the bile and ultimately the feces [4,5]. Cholesterol efflux from macrophages is the first and potentially most important step in macrophage RCT, by which intracellular cholesterol from macrophage is transferred to extracellular acceptors such as high-density lipoprotein (HDL) [4,5,6,7]. ATP-binding cassette transporters A1 and G1 (ABCA1, ABCG1) and their transcriptional factors Liver X receptor alpha (LXRα) and peroxisome proliferator-activated receptor gamma (PPARγ) are essential regulators in cholesterol efflux from macrophages [4,5,8].

Sesamin is the most abundant oil-soluble lignan in *Sesamum indicum* seeds and oil and exerts diverse pharmacological functions, including antioxidant [9], anti-inflammation [10], anti-hypertensive [11], anti-cancer [12] and cholesterol-lowering activities [13]. Dietary sesamin also efficiently inhibits the sequential development of hypertension [11], cardiovascular hypertrophy [14], and renal-, liver-, and pancreas-damage [15,16,17]. Various mechanisms are involved in sesamin-mediated health benefits such as modulation of lipid biosynthesis and oxidation [18,19], alleviation of oxidative stress [20], and induction of cell cycle arrest and apoptosis [21]. A recent report [22] revealed that the inhibitive effect of sesame-containing oil on nutritional fibrosing steatohepatitis resulted partly from the upregulation of PPARγ, a key regulator in lipid and glucose metabolism as well as in cholesterol efflux from macrophages.

As is known hypercholeslerolemia, inflammation and lipid peroxidation are key inducers of atherosclerosis. Treatment with PPARγ agonists such as rosiglitazone can efficiently prevent the occurrence and development of atherosclerosis [23]. The beneficial effects of sesamin on hypercholesterolemia, inflammation, peroxidation and PPARγ expression therefore make it a promising candidate for the prevention and treatment of atherosclerosis. Recently, Loke *et al*. reported the effect of sesamin on the atherosclerosis and found that treatment with sesamin (64 mg/kg) alleviated almost all the parameters in atherosclerotic ApoE‒/‒mice but the effect was not statistically significant [24]. We think it may be due to the low dose they used. A higher dose of sesamin may be effective for the treatment and prevention of atherosclerosis. In addition, the effect of sesamin on foam cell formation and cholesterol efflux from macrophages has not been reported up to now. In this work, we performed an *in vitro* investigation about the effect of sesamin on foam cell formation and cholesterol efflux in RAW264.7 cells. The potential mechanism of sesamin-stimulated cholesterol efflux was explored. Our results provide evidence for the utility of sesamin and sesamin-containing food like sesame oil for the prevention and treatment of atherosclerosis.

## 2. Results and Discussion

### 2.1. Sesamin Inhibits oxLDL-Induced Cholesterol Accumulation in RAW264.7 Cells

The accumulation of cholesterol-laden macrophages (foam cells) in the arterial wall is an indication of plaque-build up or atherosclerosis. Therefore, we first assessed the inhibitive effect of sesamin on intracellular cholesterol accumulation in oxLDL-elicited RAW264.7 macrophages.

**Figure 1:**
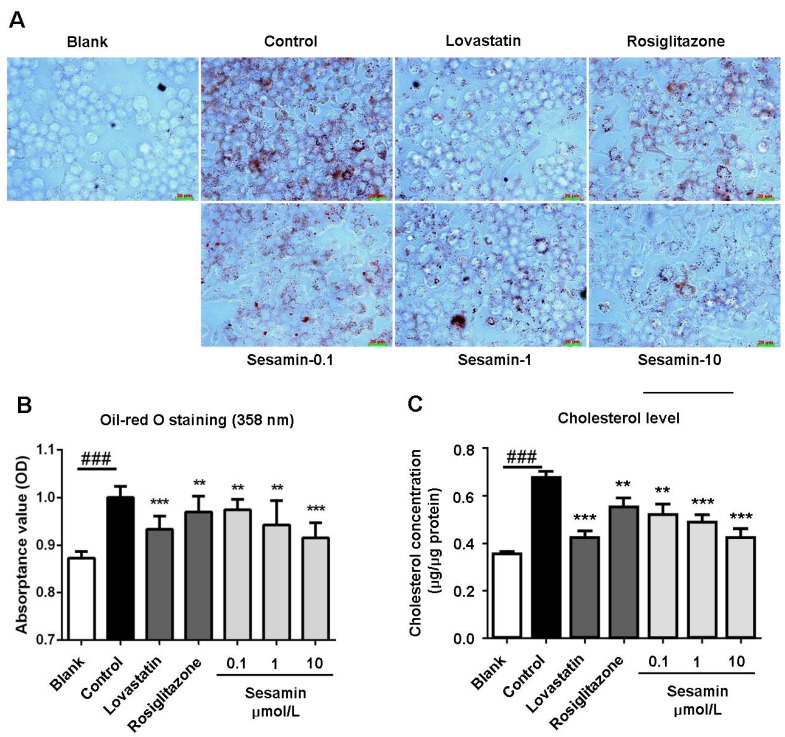
The inhibitive effect of sesamin on oxLDL-elicited lipid accumulation in RAW264.7 cells. (**A**) Typical photographs of RAW264.7 macrophages after oil-red O staining. Bar = 20 μm. (**B**) Spectrophotometry at 358 nm of RAW264.7 macrophages after oil-red O staining. (**C**) Intracellular concentration of cholesterol. RAW264.7 cells were treated with lovastatin (1 μM), rosiglitazone (1 μM) or indicated concentration of sesamin in serum-free DMEM containing oxLDL (50 μg/mL) for 24 h then subjected to oil red O staining and intracellular cholesterol quantification. Values represent mean ± SD. Results are representative of four different experiments. ^### ^*p* < 0.001 *versus* blank group, ** *p* < 0.01, *** *p* < 0.001 *versus* control group.

As shown in Figure 1, supplementation with oxLDL resulted in an enormous accumulation of cholesterol in RAW264.7 macrophages (control *vs*. blank). Treatment with sesamin significantly and dose-dependently decreased intracellular cholesterol level with an efficiency comparable to the popular hypocholesterol drug lovastatin and superior to the marketed cholesterol efflux stimulator rosiglitazone. These results suggest that sesamin can efficiently inhibit oxLDL-induced cholesterol accumulation and thus prevent foam cell formation in RAW264.7 macrophages. 

### 2.2. Sesamin Enhances Cholesterol Efflux from RAW264.7 Macrophages

**Figure 2:**
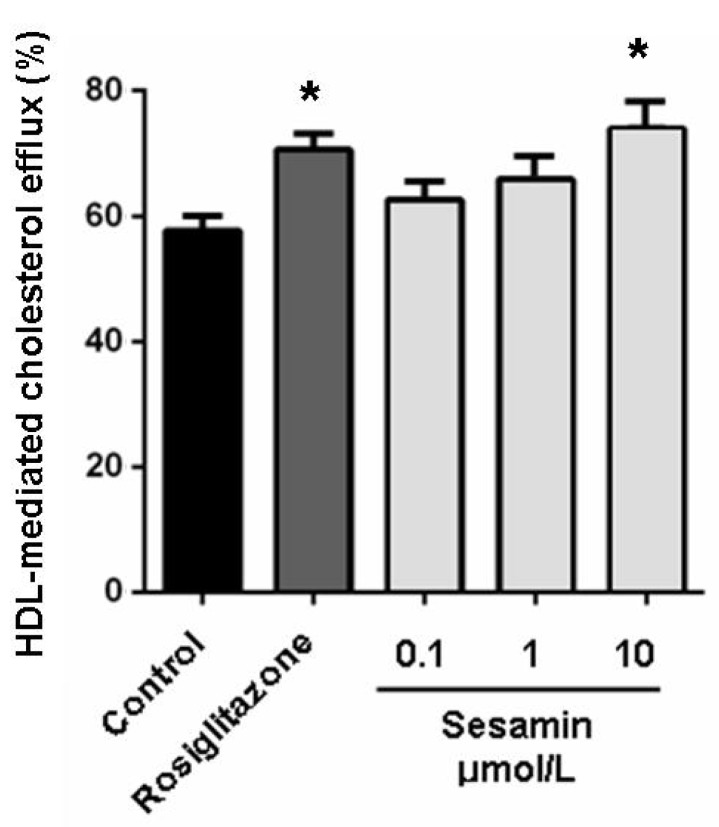
Sesamin enhances HDL-mediated cholesterol efflux from RAW264.7 macrophages. Cells were treated with rosiglitazone (1 μM) or indicated concentration of sesamin for 6 h after incubation with 25-NBD cholesterol for 24 h. Values represent mean ± SD. Results are representative of four different experiments. * p < 0.05 *versus* control group.

In our previous studies, the cholesterol-lowering potency of sesamin was weaker than that of lovastatin (27.3% *vs*. 35.4%, both at 10 μM). The comparable effectiveness of sesamin to lovastatin in decreasing oxLDL-induced cholesterol accumulation in RAW264.7 cells may thus involve another mechanism than hypolipidemic actions. Stimulation of cholesterol efflux from macrophages is a most likely mechanism adding to the hypocholeseterol acitivity of sesamin in preventing foam cell formation. As shown in Figure 2, sesamin at 10 μM significantly increased cholesterol efflux mediated by HDL with an efficiency comparable to that of rosiglitazone at 1 μM (Figure 2). This confirmed our conjecture that sesamin is an efficient stimulator of cholesterol efflux from RAW264.7 macrophages. As we can see from Figure 1 and Figure 2, sesamin is weaker than rosiglitazone in promoting cholesterol efflux at same concentration (1 μM) (Figure 2) but is more potent in inhibiting cholesterol-laden foam cell formation (Figure 1). A reasonable explanation for this contradiction is that sesamin can inhibit macrophage-derived foam cells formation through multiple mechanism such as cholesterol-lowering and cholesterol efflux-stimulating functions.

### 2.3. Sesamin Increases Transcription Of Cholesterol Efflux-Associated Genes

**Figure 3:**
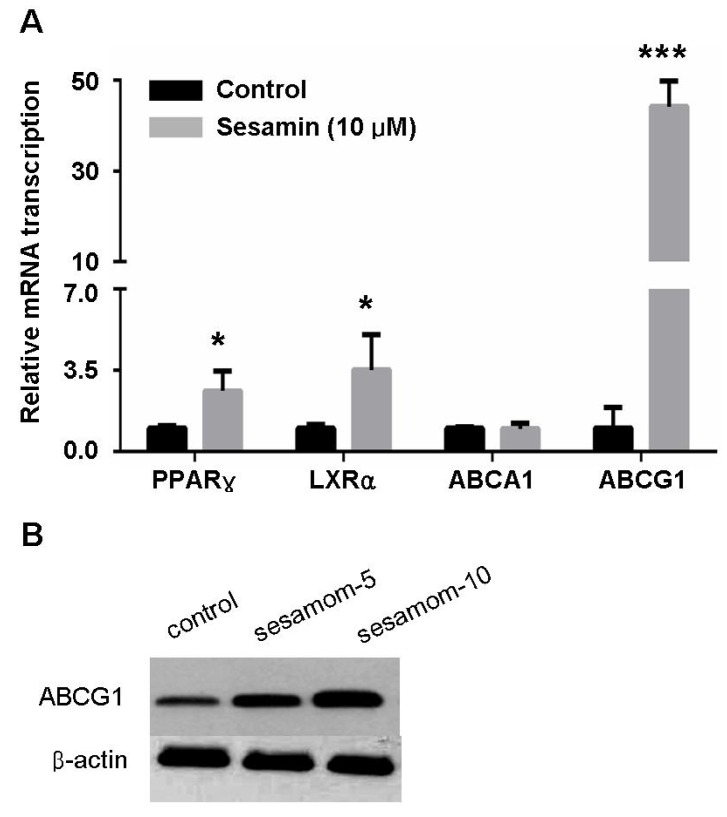
Sesamin increases expression of PPARγ, LXRα and ABCG1 in RAW264.7 macrophages. (**A**) Realtime PCR analysis for PPARγ, LXRα, ABCA1 and ABCG1. (**B**) Western blot for ABCG1. Values represent mean ± SD. Results are representative of three different experiments. * *p* < 0.05, *** *p* < 0.001 *versus* control group.

Cholesterol efflux from macrophages is regulated by various factors in which ABCA1 and ABCG1 play a pivotal role [1]. ABCA1 facilitates the efflux of cellular cholesterol to extracellular acceptors, namely, lipid-free or lipid-poor apolipoprotein A-I (ApoA-1) [4]. In contrast, ABCG1 is highly expressed in macrophages and mediates the efflux of cholesterol to HDL [4]. Realtime quantitative PCR showed that treatment with sesamin significantly increased the mRNA level of ABCG1 but not ABCA1 (Figure 3). Correspondingly, sesamin significantly enhanced cholesterol efflux mediated by HDL (Figure 2) but showed no effect on ApoA-1 mediated cholesterol efflux (data not shown). 

**Figure 4:**
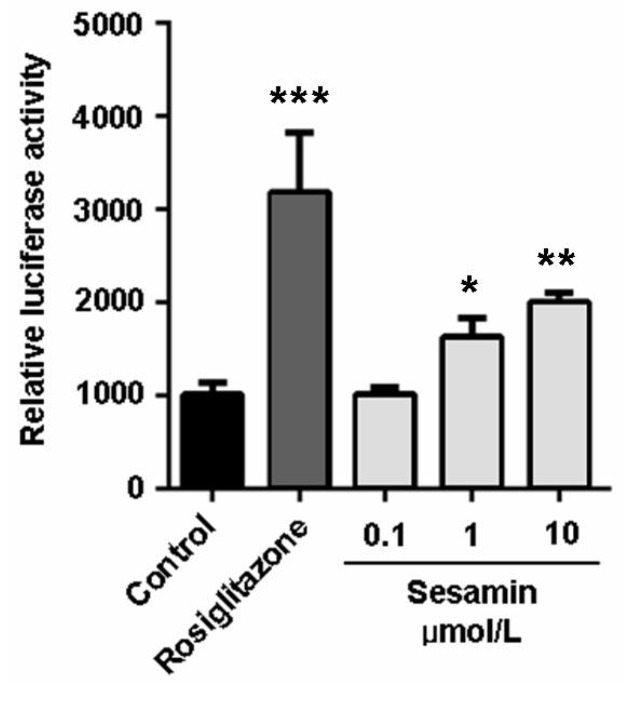
Sesamin increases transcriptional activity of pparγ. The transcriptional activity was evaluated by luciferase assay in 293T cells. Values represent mean ± SD. Results are representative of three different experiments. * *p* < 0.05, ** *p* < 0.01, *** *p* < 0.001 *versus* control group.

ABCA1 and ABCG1 in macrophages are transcriptionally regulated by ligand-dependent nuclear receptors: PPARγ-LXR pathway [4,5,8]. As previsouly reported, the induction of ABCA1 expression by PPARγ agonists is dependent on LXRs [25]. On the contrary, PPARγ agonists can also increase ABCG1 levels through an uncharacterized and LXR-independent way [25]. In this study, sesamin significantly increased the expression of PPARγ in RAW264.7 cells (Figure 3) and enhanced its transcriptional activity as determined by luciferase assay performed in 293T cells (Figure 4). It also increased the transcription of LXRα (Figure 3). However, sesamin did not increase the mRNA level of ABCA1 (Figure 3A) although it greatly enhanced the mRNA (Figure 3A) and protein (Figure 3B) levels of ABCG1. These data indicate that sesamin may stimulate cholesterol efflux from RAW264.7 macrophages through upregulation of PPARγ, LXRα and ABCG1. As for the differential modulative effect of sesamin on ABCA1 and ABCG1 expression and whether the expression of ABCG1 is regulated by LXRα-dependent or LXRα-independent pathway, more detailed investigations are needed to clarify its mechanism in the future.

### 2.4. The Cholesterol Efflux-Promoting Effect of Sesamin is Abolished by Pparγ Antagonist Gw9662

**Figure 5:**
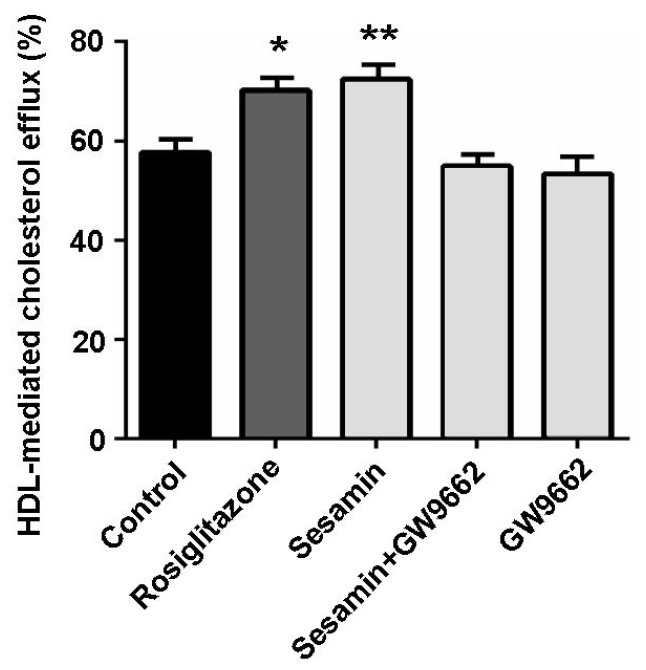
PPARγ antagonist GW9662 substantially abolished sesamin-mediated cholesterol efflux from RAW264.7 macrophages. Cells were treated with rosiglitazone (1 μM), sesamin (10 μM), GW9662 (10 μM) or sesamin + GW9662 (10 μM + 10 μM) for 6 h after incubation with 25-NBD cholesterol for 24 h. Values represent mean ± SD. Results are representative of three different experiments. * *p* < 0.05, ** *p* < 0.01 *versus* control group.

To further certificate the key role of PPARγ in sesamin-stimulated cholesterol efflux from RAW264.7 macrophages, a potent PPARγ antagonist GW9662 [26] was used. As shown in Figure 5, the cholesterol efflux-promoting effect of sesamin was substantially abolished when GW9662 was simultaneously supplemented. These results suggest that PPARγ plays an essential role in sesamin-mediated cholesterol efflux from RAW264.7 macrophages.

### 2.5. Sesamin Shows no Effect on Oxldl Uptake in Raw264.7 Macrophages

**Figure 6:**
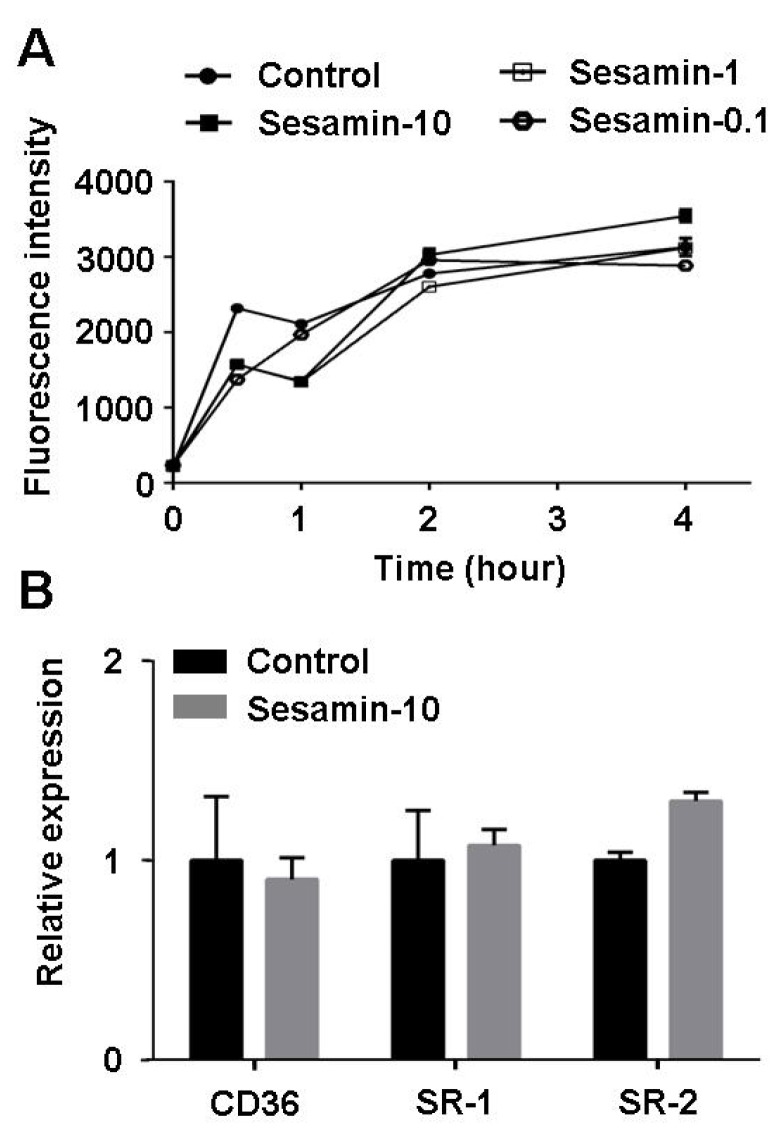
Sesamin shows no effect on cholesterol uptake by RAW264.7 macrophages. (**A**) Cholesterol uptake assay using 25-NBD cholesterol as fluorescence indicator. (**B**) Transcription levels of CD36, SR-1 and SR-2 determined by realtime quantitative PCR. Values represent mean ± SD. Results are representative of three different experiments.

The formation of foam cells is usually caused by either the uncontrolled uptake of cholesterol or impaired cholesterol efflux. To verify the effect of sesamin on cholesterol uptake, cholesterol uptake assay using NBD-cholesterol as fluorescence indicator was performed. As shown in Figure 6, sesamin showed no significant effect on either cholesterol uptake (Figure 6A) or mRNA transcription levels of cholesterol uptake-related genes CD36, SR-1 or SR-2 (Figure 6B), suggesting that the inhibitive effect of sesamin on foam cell formation does not involve in suppression of cholesterol uptake.

## 3. Experimental

### 3.1. Materials

Sesamin, lovastatin, rosiglitazone, 25-NBD cholesterol, oil red O and Dulbecco’s modified Eagle’s medium (DMEM), were procured from Sigma-Aldrich, Inc. (St Louis, MO, USA). The intracellular cholesterol assay kit was purchased from Jian Cheng Biotechnology Company (Nanjing, China). Human oxLDL and HDL were obtained from Yiyuan Biotechnologies (Guangzhou, China). A total RNA extraction reagent RNAiso Plus, a PrimeScript RT reagent kit, and a SYBR-Green PCR kit were purchased from Transgene Biotech, Inc. (Beijing, China).

### 3.2. Cell Culture and Cholesterol Accumulation Assay

RAW264.7 macrophages were originally obtained from the American Type Culture Collection (Manassas, VA, USA) and cultured in DMEM containing 10% fetal bovine serum at 37 °C under 5% CO_2_. Cells were grown to 70%–80% confluence and then treated with respective agents in indicated concentrations in serum-free DMEM containing oxLDL (50 μg/mL) for 24 h. Cells were then washed three times and subjected to oil-red O staining or cholesterol determination as described previously [27,28].

### 3.3. Cellular Cholesterol Efflux Experiments

Cellular cholesterol efflux experiments were performed using 25-NBD-cholesterol in RAW264.7 macrophages. The cells were plated in 96-well clear-bottom black plates (Costar, Corning Inc., Corning, NY, USA) at 4 × 10^4^ cells/well. Six hours later, the medium was removed and the cells were labeled with 25-NBD-cholesterol (5 µg/mL) in serum-free DMEM for 24 h in a 37 °C, 5% CO_2_ incubator. After 24 h of labeling, cells were washed twice with PBS and incubated with 200 µL serum-free DMEM containing respective agents at indicated concentrations and HDL (50 µg/mL) for an additional 6 h. Then the amounts of cholesterol in medium and cells were measured using a Tecan Infinite M1000Pro Microplate Reader, respectively (TECAN Group Ltd, Shanghai, China; excitation 485 nm, emission 535 nm). The percentage of 25-NBD-cholesterol efflux was calculated as (medium)/(medium + cell) × 100%. Each efflux assay was performed in duplicate in three experiments.

### 3.4. Quantitative Real-Time PCR

The mRNA levels of lipid metabolism-related genes were determined by real-time quantitative PCR. Total RNA extraction, cDNA synthesis and quantitative PCR assays were performed as described previously [29]. Samples were cycled 40 times using a Fast ABI-7500 Sequence Detector (Applied Biosystems, Foster City, CA, USA). ABI-7500 cycle conditions were as follows: 5 min at 95 °C followed by 40 cycles of 15 s at 95 °C, 30 s at 60 °C and 30 s at 72 °C. Cycle threshold (CT) was calculated under default settings for real-time sequence detection software (Applied Biosystems). At least three independent biological replicates were performed to check the reproducibility of the data. The gene-specific primers used for quantitative PCR are listed in Table 1.

**Table 1:** Primers used in realtime quantitative PCR analysis.

Name	Forward (5'–3')	Reverse (5'–3')
PPARγ	GCAGCTACTGCATGTGATCAAGA	GTCAGCGGGTGGGACTTTC
LXRα	AGGAGTGTCGACTTCGCAAA	CTCTTCTTGCCGCTTCAGTTT
ABCA1	CCCAGAGCAAAAAGGGACTC	GGTCATCATCACTTTGGTCCTTG
ABCG1	CAAGACCCTTTTGAAAGGGATCTC	GCCAGAATATTCATGAGTGTGGAC
CD36	CAAGCTCCTTGGCATGGTAGA	TGGATTTGCAAGCACAATATGAA
SR-1	TTAAAGGTGATCGGGGACAAA	CAACCAGTCGAACTGTCTTAAG
SR-2	TTAAAGGTGATCGGGGACAAA	AGCTGATCTTAAAAGGGTCTTG
β-actin	CCTGGCACCCAGCACAAT	GCCGATCCACACACGGAGTACT

### 3.5. 25-NBD Cholesterol Uptake Assay

Cholesterol uptake assay was performed using 25-NBD cholesterol in RAW264.7 macrophages. The cells were plated in 96-well clear-bottom black plates (Costar) at 4 × 10^4^ cells/well. Six hours later, the medium was removed and the cells were labeled with 25-NBD-cholesterol (5 µg/mL) in serum-free DMEM containing indicated concentration of sesamin or equal volume of DMSO for indicated time. Then cells were washed twice with PBS and the amounts of cholesterol in cells were measured using a Tecan Infinite M1000Pro Microplate Reader (TECAN Group Ltd.; excitation 485 nm, emission 535 nm). Each uptake assay was performed in duplicate in three experiments.

### 3.6. Luciferase Assay

Luciferase assay was performed in 293T cells. Cells were transiently transfected with PPARγ expression vector and DR-1 luciferase reporter vector by lipo2000 (Invitrogen, Shanghai, China) according to the manufacturer’s instrument. At 6 h after transfection, the transfection mixture was replaced with fresh medium containing the appropriate agonist. Luciferase assays were performed after 24 h using luciferase assay kit (Promega, Beijing, China) according manufacturer’s instruction.

### 3.7. Statistics Analysis

The results were expressed as mean ± SD. A one-way analysis of variance (ANOVA) was done using SPSS13.0 software. Significance was accepted at *p* < 0.05.

## 4. Conclusions

This work demonstrated that sesamin isolated from *Sesamum indicum* is able to inhibit cholesterol accumulation elicited by oxLDL and stimulate cholesterol efflux from RAW264.7 macrophages. Upregulation of PPARγ-LXRα-ABCG1 pathway may involve in the promoting effect of sesamin on cholesterol efflux. These results provide *in vitro* evidence that sesamin and sesamin-containing food like sesame oil can be favorably used for the prevention and treatment of atherosclerosis.
